# An Interactive Session on Nutritional Pathologies for Health Professional Students

**DOI:** 10.3390/healthcare3030519

**Published:** 2015-07-07

**Authors:** Joshua DeSipio, Sangita Phadtare

**Affiliations:** 1 Department of Medicine, Gastroenterology/Liver Diseases Division, Cooper Medical School of Rowan University, Camden, NJ 08103, USA; E-Mail: DeSipio-Joshua@CooperHealth.edu; 2 Department of Biomedical Sciences, Cooper Medical School of Rowan University, Camden, NJ 08103, USA

**Keywords:** nutrition, nutritional pathology, interactive session, peer teaching

## Abstract

Various studies have emphasized the need to improve the nutrition training of health professionals, which will help them to provide optimal patient care. Nutrition-based interactive sessions may serve as an efficient approach to instigate an interest in nutrition among the students. Here we report the reception and effectiveness of a nutrition-pathology based interactive activity that we designed and implemented in the gastroenterology course given to the second year students at our medical school. The activity involved team work, individual accountability and peer-teaching. Nutrition pathology case stems (Kwashiorkor, vitamin B-12 deficiency, zinc deficiency and zinc-induced copper deficiency) were posted on the course website for the students to read before the session. At the start of the session, all the groups (each made up of four members) took a pre-quiz. Each student was then given an information sheet describing one case. Each group discussed the four cases with students acting as the “teacher” for the case assigned to them. A post-quiz was administered to the groups to assess acquisition of knowledge as well as in-depth thinking about the nutrition aspects discussed. The efficacy of the session measured by pre (39% questions correctly answered in total) and post-quizzes (96% questions correctly answered in total) and the overwhelmingly positive student feedback indicated that the session was highly effective. Ninety-five percent of students thought that the session demonstrated the clinical relevance of nutrition, while 98% students found the peer teaching to be engaging.

## 1. Introduction

Nutrition plays a significant role in wide-spread diseases including those caused by deficiency of macronutrients and micronutrients. The 2011 Association of American Medical Colleges (AAMC) report emphasizes that diet and inadequate exercise are among the main causative factors that lead to more than half of premature morbidity and mortality. Thus, tomorrow’s physicians will be required to have advanced knowledge, skills, and attitudes in several nutrition-based domains [1]. Results of a 2010 national survey showed that less than one third of medical schools in the United States meet the minimum 25 required hours set by the National Academy of Sciences [2]. Other studies also reported that most health care professionals do not receive adequate nutrition training and do not possess the expertise to provide adequate nutrition counseling to their patients [3,4]. These observations accentuated the need for improving the nutrition training of health professionals which will help them in providing optimal patient care [5]. This has prompted the current increased awareness about nutrition education for health profession students in both preclinical as well as clinical years.

The Cooper School of Rowan University is a new medical school that utilizes lectures, laboratories, and active learning (problem-based-solving) exercises through the preclinical years. Instead of having a dedicated nutrition course, nutrition is integrated in various courses such as Fundamentals, Lifestages and Hematology-Oncology (first year courses), Gastroenterology and Women’s health (second year courses). This allows the students to understand nutrition as an underlying principle in context of various body systems and corresponding disorders. The Gastroenterology (GI) course includes the overview of vitamins and minerals. Here we describe the reception and efficacy of an interactive activity on nutritional pathology that was introduced in this course. Inclusion of nutrition-based interactive sessions in the curriculum gives the students an opportunity to learn to critically think about nutrition [6]. Active learning approaches are proving to be very effective for improving knowledge retention, communication skills, and self-directed learning and lead to deeper understanding of the material [7,8,9,10]. A comprehensive compilation of a variety of active learning methods that can be used by medical educators was published recently [11]. We created an interactive activity that included combination of elements from different active learning methods to suit our goals. This activity built upon the aspect of digestion and absorption of nutrients that was taught in the GI course. The goals of this activity were to (i) incorporate new active learning methods in the curriculum, which focus on teamwork as well as individual accountability, (ii) improve student appreciation of clinical relevancy of nutrition, (iii) encourage thinking about nutrition using unusual, real life cases. Our overall objective for this study was to instigate an interest in nutrition among the medical students.

## 2. Experimental Section

### 2.1. Cases Selected for the Activity

The cases were based on (i) Kwashiorkor, (ii) vitamin B-12 deficiency, (iii) zinc deficiency and (iv) zinc-induced copper deficiency. All the cases presented were based on real-life scenarios. In each case the underlying nutrient deficiency was not immediately apparent and had required the respective attending physicians to carry out in-depth analysis of the data and “out-of-the-box” thinking to come up with the correct diagnosis. During the session, the students were given information about clinical presentation, patient history, laboratory data, underlying cause, physiology and treatment options for each case. Important concepts and terminologies were highlighted in bold or were underlined. Conclusions were provided at the end. Highlights of each case are presented in Table 1.

**Table 1:** Highlights of the nutritional pathology cases used in the session.

Deficiency	Case Highlights	Conclusions
Kwashiorkor: due to insufficient protein intake or absence of one or more of the essential amino acids in the diet.	Rapid development of Kwashiorkor in a baby who was weaned of breast feeding and put on a simple diet. The symptoms ranged from skin pallor to edema and susceptibility to infections.	Diet analysis showed that the baby’s diet had deficiency (84%) of a single essential amino acid. Young children are very susceptible to deficiency of a single essential amino acid even though it may not be a statistically dramatic deficiency [12].
Vitamin B-12 deficiency	Patient reported feeling dizzy when he gets up and had uncontrolled shakes and twitches in his legs. He has been in and out of hospital for treatment for a mild stroke.	It is not usual to ask your patient to walk, but in this case that helped the diagnosis. When asked to walk, the patient wobbled and walked with a slapping gait, suggesting a problem with the nerves. Vitamin B-12 deficiency may result in anemia and neurological symptoms. Even though vitamin B-12 is required in minute amounts, problems in its absorption can lead to dire consequences [13].
Zinc deficiency	Patient reported with diarrhea; developed skin lesions all over body.	Case illustrated how zinc deficiency can develop due to a variety of reasons such as a genetic deficiency, lack of zinc in the TPN given to the hospitalized patients, recurrent diarrhea for a prolonged time, bariatric surgery, or chemotherapy patients whose nutrient intake is not optimal [14–16].
Zinc-induced copper deficiency	A patient with previous partial gastrectomy has fatigue, numbness of hands and abnormal sensation in hands and feet (anemia and myelopathy). Patient takes supplements which are below their upper limit (UL) for toxicity.	Megadoses of zinc may interfere with copper absorption leading to copper deficiency, as these two share a common protein for their absorption. Overall digestion and absorption is affected in this patient due to the removal of part her stomach. The changed absorption modified the UL of zinc for her and proved to be toxic [17].

### 2.2. Before the Session

A brief description of the main goals and format of the one hour session was given in the introduction lecture of the course and detail instructions were posted on the course website. The stems containing clinical presentation of the four cases were posted on the course. The students were required to read these stems before they came to the session as preparation for the session. The students were not required to carry out research about the cases, but were asked to think about what the underlying problem could be based on the nutrition concepts they had learned in various courses.

### 2.3. During the Session

The activity was carried out in a single lecture hall with two faculty facilitators who are the course directors of the GI course. This obviated the need for participation of a number of faculty facilitators who are either experts on nutrition or need to be trained for the session. Students were randomly assigned into groups of four. Groups were physically separated from each other in the lecture hall to allow discussions without noise disturbance from neighboring groups.

First, the students took a pre-quiz as a group. Each student within a group was assigned to learn about one of the four nutrition-based cases. Each case was assigned a color: blue (Kwashiorkor), green (vitamin B-12 deficiency), yellow (zinc deficiency), purple (zinc-induced copper deficiency). Although it is not essential to assign colors to the cases, it facilitated easy and rapid distribution of four cases, which was particularly helpful given the time constraints of the session. After the pre-quiz, each student received a one and half pages long colored sheet with the case assigned to him/her. Each of the sheets contained the stem of the case along with above-mentioned information. Students were given about 15–20 min to read the individual cases. Then the team had 20–25 min to go through all of the material together, in which each student discussed his/her case with the other three students. The students were encouraged to ask thought-provoking questions to their teammates to facilitate discussion. Students were allowed to research resources during the session (except during quizzes) and also ask faculty for help. The two faculty facilitators were going around the lecture hall throughout the session to provide help as needed and also ask questions to assess the effectiveness of peer-teaching. At the end, the students took a post-quiz as a group.

### 2.4. Pre and Post-Quizzes

Both the quizzes consisted of eight multiple choice questions and were comparable. The students were given 8 min for each of the quizzes. The quizzes contained both fact-based and comprehension-based questions. The questions were consistent with the NBME style exam questions and most were case-based. Examples of two such questions are shown in Box 1. The answers to the quiz questions were posted in the course website after the quizzes were graded. The activity counted towards 3% of their course grade.

### 2.5. Activity Evaluation Surveys

Students evaluated this activity after its completion. Out of the 62 students who participated in the activity, 55 students chose to complete the surveys. The primary purpose of collecting student comments was to evaluate the activity and make changes to improve for the future implementation. As this was a new activity introduced in this course, it was especially imperative to evaluate it with respect to its learning objectives. The survey contained two Likert scale questions: (i) if the nutrition pathology session demonstrated the clinical relevance of nutrition and (ii) and if students found the peer-to-peer teaching method to be engaging. The third question asked for comments on the usefulness of the activity and if the students found the cases to be intriguing. The protocol for administering the anonymous paper surveys was as per the guidelines set by Rowan Institutional Review Board. The authors received approval from the Human Subjects Protection Program Institutional Review Boards (IRB) at the Rowan University on 2 December 2014 (project ID: Pro2014000201). Students at our medical school are routinely required to complete evaluation surveys for various components of all the courses, which are then provided to respective faculty. The students were informed that the evaluation of this activity was voluntary and anonymous and did not affect their grades in any way. No personal identification markers were used in the survey.

Box 1 **Examples of quiz questions**

Comprehension-based question:A 75-year old man presents with a several week history of fatigue. He shows a severe pallor. Neurological examination revealed short-term memory loss and a decreased sense of vibration in the legs. Blood test results showed his hemoglobin to be 3.8 g/dL (normal: 14–18 g/dL), mean corpuscular volume to be 105 μM^3^ (normal: 82–92 μM^3^). His serum vitamin B-12 was 9 pg/mL (normal: >200 pg/mL). Which one of the following is the most likely cause of vitamin B-12 deficiency in this patient?
Bacterial destruction of vitamin B-12Increased production of acidDecreased production of pancreatic proteasesDefect in the absorption of fat
Fact-based question:Acrodermatitis Enteropathica develops due to
lack of sufficient amount of zinc in the diet.lack of sufficient amount of copper in the diet.defect in a protein involved in the absorption of copper.defect in a zinc transporter.

## 3. Results

**Figure 1:**
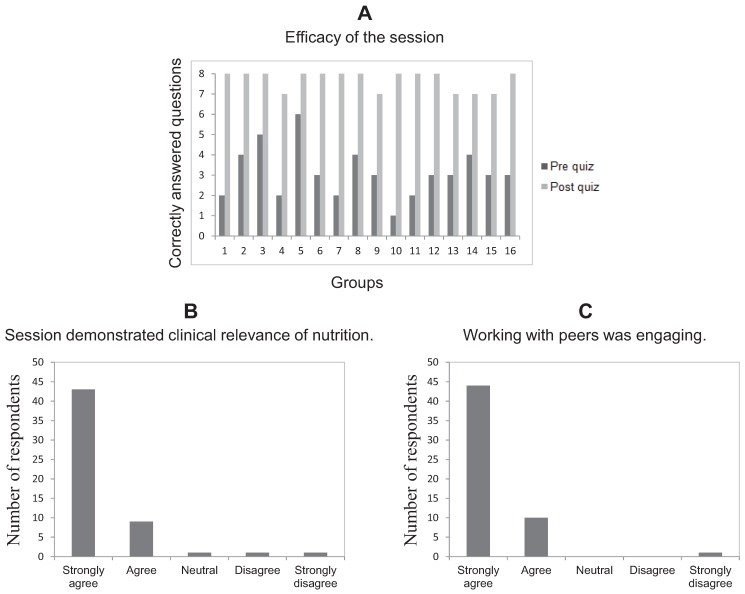
(**A**) Measurement of the efficacy of the session based on correctly answered questions in pre and post quizzes. Note that the combination of group numbers and corresponding numbers of correctly answered questions is different from the actual combination. This has been done to further protect the anonymity of the subjects (for example, the data presented for group 1 in the figure may be from any group numbered 1–16 in the actual session). Quantitative representation of the (**B**) demonstration of the clinical relevance of nutrition and (**C**) engaging nature of working with peers as measured by the students’ evaluation of the activity. The students were asked to complete a survey which contained two Likert scale questions (five choices are shown) regarding these two points.

The interactive session required the students to work in groups and be responsible members of their groups. All students participated actively in the session. A number of students commented that they were not able to identify the underlying problem in the cases posted before the session. They thus appreciated how “out-of-the-box” thinking was important for diagnosis of these cases. The efficacy of the session as measured by the differences between pre and post-quiz scores demonstrated that the session was highly effective (Figure 1A). Both quizzes included eight multiple choice questions, two on each of the four cases. Each quiz contained six case-based and two fact-based questions. The case-based questions required higher level reasoning. In the pre-quiz, 14 out of 16 teams answered half or more than half of the questions incorrectly. Thus, 39% questions were answered correctly in total in the pre-quiz. No bias was observed with respect to fact-based *versus* case-based questions in the correctly answered questions. On the other hand, 11 out of 16 teams answered all of the eight questions correctly in the post quiz, while the remaining five teams answered only one question incorrectly. Thus, 96% questions were answered correctly in total in the post-quiz. Out of the total of five incorrectly answered questions among all the groups, two questions were fact-based (zinc deficiency) and three questions were case-based (vitamin B12 deficiency and Kwashiorkor). The students did very well (more than 80% students answering questions correctly) on the questions based on the session materials in the final course exam.

The students’ evaluations of the session were overwhelmingly positive. Quantitative representation of the responses received to the two Likert scale questions in the survey is given in Figure 1B,C. To the question, “if the session demonstrated clinical relevance of nutrition”, 43 out of 55 students (78.18%) chose “strongly agree”, while nine students (16.36%) chose “agree”. One student each chose “neutral”, “disagree” and “strongly disagree” to this question. To the question if “working with peers was engaging”, 44 out of 55 students (80%) chose “strongly agree”, while 10 students (18.18%) chose “agree”. Only one student chose “strongly disagree”. All of the 50 comments received for the open ended question, “please comment on the usefulness of the Nutrition pathology session. Did you find the cases intriguing?” were positive. The main aspects liked by the students were (i) engaging and intriguing nature of the cases (ii) real life cases, (iii) teaching to and learning from their peers; (iv) no extensive pre-session work and efficient covering of four cases, (v) logical thinking and interactive way of learning and (vi) the session tied together everything they had learned. Examples of some representative comments include, “This was wonderful. Speaking with peers helped learn the material better than if we had just read it on our own.”, “Yes, these cases were fascinating and engaging. I learned a great deal from them.”, “Helpful-really nice learning from my peers and having time to talk through the cases, ask questions.”, “Yes, used logical thinking and even learned new material.”, and “Very useful way to efficiently cover four cases”. One student suggested that a five minute debrief would be helpful and another suggested that more time be given for the session instead of one hour. A third student found the session useful and cases intriguing, but did not feel that much ground was covered. We will consider these suggestions for the next year’s session.

## 4. Discussion

CMSRU is a new medical school, thus one of the goals for creating this activity was to introduce new interactive approaches in our curriculum which focus on team work as well as require individual accountability. We wished to create an activity that builds on the nutrition, biochemistry and physiology content taught in the Gastroenterology course and makes the students intrigued about nutrition by discussing real life nutrition pathology cases. The success of this activity was heavily dependent on peer-teaching. Interest in peer-teaching (or peer-assisted learning/PAL) is growing in the field of medical and allied health education. Existing evidence suggests that peer teaching benefits the students academically and professionally. The success of PAL lies in the fact that participating students share cognitive congruence as well as social congruence. Inclusion of peer-teaching in undergraduate medical education helps the students to bridge the gap between being a university student and being a clinician [18,19,20]. Developing teaching skills and using these skills for application and translation of medical knowledge is important for the progress of all medical trainees [21]. The results indicate the effectiveness of this interactive activity in student acquisition of knowledge as well as enhancement of comprehension, which was likely facilitated by interaction with peers. As mentioned above, 98% of students liked the peer teaching aspect of the session.

Several points contributed to the success of this activity. The cases chosen were real-life cases, which made the exercise very relevant to the medical students. A vast majority of students thus agreed that the activity demonstrated clinical relevance of nutrition. As the underlying nutrient deficiencies were not immediately obvious, the cases captured the students’ interest and also demonstrated to them that nutrition can be very complex. The short length of the session contributed towards making it highly efficient with respect to time. Length of the material (~one and half pages per case) was kept the same for each case and was appropriate for the duration of the session. Cases were also similar with respect to complexity. As the cases were built upon biochemistry, nutrition and physiology taught in the course, the exercise provided an opportunity for integration of various topics learned in the course. As important points were highlighted throughout the materials and questions were provided for discussion, students did not require additional guidance during the session. The quiz questions assessed student skills in both recalling facts as well as higher-level thinking. The activity was facilitated by only two faculty members in a large lecture hall; this obviated the need for training additional faculty in nutrition and acquiring multiple small rooms.

We would like to use this format to include examples of other nutrient deficiencies. However, we will have to create another session for this purpose. One limitation of this activity is the number of cases that can be included, which is determined by the size of the groups. Groups of four or at the most five students work better; thus, four–five cases can be discussed. Assigning more than one deficiency per student may not work well as it will require the students to learn a large volume of material and teach it to their peers. This activity was one hour long; we felt that an hour and half long session may work better for this amount of material.

As mentioned above [11], there are a variety of active learning methods being used for medical education. Team-based (TBL) and problem-based learning (PBL) are most commonly used cooperative learning methods in the preclinical years. Team-based learning involves small-group discussion facilitated by an instructor, wherein teams solve clinical problems and receive immediate feedback on performance. The first phase of TBL involves pre-class preparation by the learners. In PBL, the group works through a case stem presented to them with additional information released in a time wise manner. Educators choose the most appropriate method to achieve the desired learning outcome while considering their available resources. We designed this session based on the above stated goals, availability of time, trained faculty facilitators and resources. This activity does not need extensive preparation as opposed to that required for TBL and PBL [11] and also has a more stringent dependence on individual student participation. Although this activity was primarily developed for pre-clinical medical students, it may also be useful for other health professional or undergraduate students studying nutrition.

## 5. Conclusions

This session was built upon the aspect of digestion and absorption of nutrients that was taught in the GI course and also revisited certain nutrition aspects taught in various courses. It thus helped to achieve integration across the curriculum. It also improved the quality and effectiveness of the GI course for the students in that it provided an opportunity to them to think about nutrition from a clinical point of view and discuss it with their classmates. This form of active learning likely enhances the retention of this material. We think that we were successful in instigating an interest in nutrition among the students and hope that this will be useful to them in their clinical practice in future.

## References

[1] Kushner R.F., van Horn L., Rock C.L., Edwards M.S., Bales C.W., Kohlmeier M., Akabas S.R. (2014). Nutrition education in medical school: A time of opportunity. Am. J. Clin. Nutr..

[2] Adams K.M., Kohlmeier M., Zeisel S.H. (2010). Nutrition education in US medical schools: Latest update of a national survey. Acad. Med..

[3] Kris-Etherton P.M., Akabas S.R., Bales C.W., Bistrian B., Braun L., Edwards M.S., Laur C., Lenders C.M., Levy M.D., Palmer C.A. (2014). The need to advance nutrition education in the training of health care professionals and recommended research to evaluate implementation and effectiveness. Am. J. Clin. Nutr..

[4] Frenk J., Chen L., Bhutta Z.A., Cohen J., Crisp N., Evans T., Fineberg H., Garcia P., Ke Y., Kelley P. (2010). Health professionals for a new century: Transforming education to strengthen health systems in an interdependent world. Lancet.

[5] Darnton-Hill I., Samman S. (2015). Challenges and opportunities in scaling-up nutrition in healthcare. Healthcare.

[6] Phadtare S., Galt G., Brodsky B. (2014). Active learning approaches for nutrition education in the medical school curriculum. Med. Sci. Edu..

[7] Phadtare S., Abali E., Brodsky B. (2013). Over the counter drugs (and dietary supplement) exercise: A team-based introduction to biochemistry for health professional students. Biochem. Mol. Biol. Educ..

[8] Abali E.E., Phadtare S., Galt J., Brodsky B. (2014). An online guided e-journal exercise in pre-clerkship years: Oxidative phosphorylation in brown adipose tissue. Biochem. Mol. Biol. Educ..

[9] Thistlethwaite J.E., Davies D., Ekeocha S., Kidd J.M., MacDougall C., Matthews P., Purkis J., Clay D. (2012). The effectiveness of case-based learning in health professional education. A BEME systematic review: BEME Guide No. 23. Med. Teach..

[10] Currey J., Oldland E., Considine J., Glanville D., Story I. (2015). Evaluation of postgraduate critical care nursing students’ attitudes to, and engagement with, Team-Based Learning: A descriptive study. Intensiv. Crit. Care Nurs..

[11] Wolff M., Wagner M.J., Poznanski S., Schiller J., Santen S. (2015). Not another boring lecture: Engaging learners with active learning techniques. J. Emerg. Med..

[12] Stevenson N. (2006). Personal communication.

[13] Sanders L. Think like a doctor: The wobble solved. http://well.blogs.nytimes.com/2013/03/01/think-like-a-doctor-the-wobble-solved/?ref=healthGrandRoundscase.

[14] Saritha M., Gupta D., Chandrashekar L., Thappa D.M., Rajesh N.G. (2012). Acquired Zinc deficiency in an adult female. Ind. J. Dermatol..

[15] Bae-Harboe Y.S., Solky A., Masterpol K.S. (2012). A case of acquired Zinc deficiency. Dermatol. Online J..

[16] Chun J.H., Baek J.H., Chung N.G., Kim J.E., Cho B.K., Park H.J. (2011). Development of bullous acrodermatitis enteropathica during the course of chemotherapy for acute lymphocytic leukemia. Ann. Dermatol..

[17] Kumar N. (2006). Copper deficiency myelopathy (Human Swayback). Mayo Clin. Proc..

[18] Yu T.C., Wilson N.C., Singh P.P., Lemanu D.P., Hawken S.J., Hill A.G. (2011). Medical students-as-teachers: A systematic review of peer-assisted teaching during medical school. Adv. Med. Edu. Pract..

[19] Schmidt H.G., Moust J.H. (1995). What makes a tutor effective? A structural-equations modeling approach to learning in problem-based curricula. Acad. Med..

[20] Ten Cate O., Durning S. (2007). Dimensions and psychology of peer teaching in medical education. Med. Teach..

[21] Ologunde R., Rabiu R. (2014). Students as teachers: The value of peer-led teaching. Perspect. Med. Edu..

